# The impact of Information and Communication Technology (ICT) on learning outcomes in early childhood and primary education: a meta-analysis of moderating factors

**DOI:** 10.3389/fpsyg.2025.1540169

**Published:** 2025-06-18

**Authors:** Zuo Ruijia, Li Wenling, Zhang Xuemei

**Affiliations:** ^1^Department of Preschool Education, School of Music and Dance, Xihua University, Chengdu, China; ^2^School of Education Science, GuangXi Minzu Normal University, Chongzuo, Guangxi, China

**Keywords:** information and communication technology, early childhood education, primary education, student learning and development, meta-analysis

## Abstract

The role of Information and Communication Technology (ICT) in fostering the learning and development of young children and primary school students has become a pivotal focus in contemporary education. This study adopts a meta-analytic approach to systematically synthesize and evaluate findings from 30 recently published studies on the use of ICT in early childhood and primary education. The analysis reveals that ICT has a significant positive effect on student learning, particularly in enhancing language skills (effect size = 0.24) and subject knowledge acquisition (effect size = 0.59). Additionally, the analysis highlights the moderating effects of variables such as intervention duration and application type, emphasizing the need for context-specific implementation strategies. A random-effects model was employed to account for between-study variability, providing robust empirical evidence to inform the design and application of ICT in basic education. The study further recommends that future research prioritize the development of tailored digital resources, the evaluation of long-term impacts, and the exploration of contextual adaptability to fully realize the potential of ICT in enhancing the learning and development of young children and primary school students.

## 1 Introduction

In the context of globalization and digitalization, Information and Communication Technology (ICT) has emerged as a cornerstone of modern education systems, particularly in early and primary education, where its application has garnered increasing attention (Lawrence and Tar, 2018; Zafar, 2019). Governments and educational institutions worldwide have implemented policies to promote the deep integration of ICT into basic education. For instance, the European Union adopted the “2021–2027 Digital Education Action Plan,” which prioritizes enhancing digital literacy and embedding ICT into educational practices (European Commission, 2020; Digital Education Action Plan (2021–2027)—European Education Area, 2020). Similarly, the “Every Student Succeeds Act (U.S. Congress, 2015)” in the United States underscores the role of technology in facilitating personalized learning and improving instructional efficiency (Yang et al., 2021). In New Zealand, the “Digital Technologies Curriculum,” launched by the Ministry of Education in 2017, highlights the importance of early exposure to technology to prepare children for a digital future (Fox-Turnbull, 2019; Reinsfield, 2020). Meanwhile, in China, the Ministry of Education launched the “Education Informatization 2.0 Action Plan (2018)” aims to achieve comprehensive ICT integration in teaching, learning, and digital campus construction, alongside significant advancements in digital literacy for both teachers and students, supported by the large-scale development of educational big data (Yan and Yang, 2020). Additionally, the “14th Five-Year Plan for Education Informatization (2021)” emphasizes deepening ICT integration into basic education by advancing ICT-enhanced curriculum development, expanding the use of high-quality digital resources, and promoting online learning platforms (Poo, 2020).

Driven by policy initiatives, the integration of ICT into basic education has advanced significantly. However, its application in early childhood education remains contentious. Some researchers argue that early exposure to technology during preschool years may potentially disrupt children’s social skills, attention span, and creative thinking (Bukhalenkova et al., 2021; Zomer and Kay, 2018), while others have found excessive screen time to negatively impact cognitive development. At the same time, parents express concerns that early use of technology might adversely affect children’s vision and behavioral habits (Rutland and Killen, 2017). Nevertheless, as digital technology continues to exert profound influence on the educational landscape, the academic focus has shifted from skepticism to exploration: how to effectively design and utilize ICT to foster the development of young children and primary school students (Lavrenova et al., 2020).

Existing research has examined the value of ICT in basic education from diverse perspectives. For instance, Lin and Lin (2018) explored ICT use in primary schools and found that it significantly improved students’ reading and mathematics performance while enhancing their learning motivation and classroom engagement. Despite the growing body of literature emphasizing the positive effects of ICT on academic outcomes, recent research also raises concerns about its potential adverse or neutral effects, particularly among preschool and primary school children. Excessive screen time and poorly designed digital content have been shown to contribute to reduced attention spans, cognitive fatigue, and even developmental delays. For example, Duch et al. (2013) found that preschoolers exposed to frequent screen media exhibited lower attention regulation and executive functioning scores. Similarly, Meng et al. (2018) observed that primary students with high daily ICT usage demonstrated decreased school engagement and lower performance in reading and problem-solving. Tugtekin and Odabasi (2022) further argued that the fast-paced, overstimulating nature of some interactive applications may impose excessive cognitive load on young learners, thereby hindering deep information processing and retention. Moreover, studies such as Madigan et al. (2019) suggest a dose–response relationship between screen time and weaker outcomes in early language development, memory, and executive functioning. These findings highlight the importance of distinguishing between ICT’s effects on academic achievement and its broader impact on cognitive and attentional processes, such as self-regulation and sustained focus. A balanced and multidimensional review of both beneficial and detrimental outcomes is therefore essential for a comprehensive and evidence-based understanding of ICT’s role in early education. At the same time, a substantial body of research continues to affirm the educational potential of ICT when thoughtfully implemented. Scherer et al. (2019), through quasi-experimental studies, demonstrated that educational software and online resources can effectively enhance cognitive development, especially in areas such as problem-solving and critical thinking, while also supporting social interaction skills. Verhoeven et al. (2020) highlighted the positive impact of ICT on preschool and early primary children’s language acquisition and literacy. In parallel, Kolić-Vehovec et al. (2020), reported that well-integrated ICT practices in classrooms fostered both academic performance and student engagement. Nevertheless, some studies caution that unregulated or poorly designed technology use may distract learners, foster dependency, and increase cognitive overload (Clemente-Suárez et al., 2024; Masood et al., 2020). Other findings suggest that the educational effectiveness of ICT tools often hinges on teachers’ digital literacy and pedagogical strategies. For instance, Neumann (2018) emphasized that while tablets can support early literacy development, their benefits are highly dependent on usage quality and duration. Similarly, López-Escribano et al. (2021) found no significant improvement in early reading skills among preschoolers using e-books compared to print books, with lower comprehension outcomes observed in digital settings. Overall, although the research base is expanding, findings remain somewhat fragmented, underscoring the need for systematic synthesis and contextual analysis (Hare et al., 2024; Kareva, 2024).

Given the growing significance of ICT in preschool and primary education, there is a pressing need for a comprehensive meta-analysis to synthesize existing research and evaluate its actual impact. Meta-analysis offers a robust methodological approach to address the limitations of individual studies, providing policymakers with evidence that is both generalizable and actionable (Parr et al., 2019). This study seeks to fill this research gap by systematically assessing the effects of ICT on early childhood and primary education. The findings provide empirical insights to inform the optimized design and implementation of ICT in educational contexts, offering both theoretical foundations and practical recommendations for advancing the digital transformation of basic education.

This study addresses this gap by conducting a meta-analysis of 30 recent studies, systematically evaluating the impact of ICT on early childhood and primary education. By synthesizing existing evidence, this research offers a comprehensive overview of ICT’s actual effects across different educational settings and identifies key moderating factors, such as intervention duration and ICT application type. The novelty of this study lies in its ability to aggregate findings across diverse contexts and provide empirical insights into the conditions under which ICT is most effective in supporting student learning and development. This research not only advances academic discourse by offering a unified view of ICT’s impact but also provides actionable evidence to guide policy and practice. The findings will help policymakers and educators design more effective, context-specific ICT interventions and contribute to the ongoing digital transformation of education.

## 2 Research object

A review of existing meta-analyses examining the impact of technology on young children and primary school students reveals two significant limitations. First, most studies tend to focus on a single educational level, such as primary or higher education, without addressing the developmental continuum between early childhood and primary education—two stages that are pivotal for children’s cognitive, emotional, and social development. Second, prior research often concentrates on specific subject areas, such as mathematics or language, overlooking the broader, cross-disciplinary effects of ICT. Furthermore, while ICT has shown promise in promoting personalized learning and increasing student engagement, its effectiveness is contingent on various factors, including the duration of interventions and the types of technologies employed issues that have yet to be systematically explored.

In recent years, the integration of technology into early childhood and primary education has become increasingly widespread. Research indicates that when appropriately incorporated into teaching practices, technology can serve as a powerful tool to engage young learners in meaningful activities, thereby fostering enhanced learning outcomes and supporting developmental progress (Lawless and Pellegrino, 2007). This study seeks to extend this body of research by identifying the specific predictors that influence the effectiveness of technology in shaping the learning outcomes of young children and primary school students.

From the perspectives of biology, neuroscience, and psychology, early childhood and primary education are critical periods for the development of fundamental neural connections, cognitive abilities, and social–emotional skills (Johnstone et al., 2022; Sinclaire-Harding et al., 2018). During these formative stages, ICT plays a pivotal role by offering not only abundant learning resources and diverse educational experiences but also multisensory stimuli that enhance learning outcomes (Lawrence and Tar, 2018). Moreover, ICT supports personalized learning by providing interactive platforms and tailored resources that cater to the unique developmental needs of each child, thereby optimizing their learning potential (Major et al., 2021). Educators can leverage ICT tools—such as multimedia resources, interactive learning platforms, and virtual reality technologies—to enrich instructional content and foster deeper engagement (Fonseca and García-Peñalvo, 2019). For instance, interactive whiteboards and educational apps enable students to comprehend complex concepts through gamified, interactive approaches (Reguera and Lopez, 2021). Thus, the integration of ICT in early childhood and primary education represents a highly innovative and significant area of research, with the potential to transform teaching and learning in these crucial developmental stages (Akyar et al., 2024; Ihmeideh and Al-Maadadi, 2018).

Against this backdrop, this study employs a meta-analytic approach to systematically examine the impact of ICT on the learning and development of young children and primary school students. The specific objectives are as follows:

1. To synthesize and integrate existing literature evaluating the impact of ICT on young children and primary students, with attention to the distribution of studies across geographic regions and publication years.
2. To explore the influence of various predictors, such as subject domain, application type, and intervention duration, on the effectiveness of ICT.

To achieve these objectives, a meta-analytic method is employed, using effect size as a measure to assess the overall impact and heterogeneity of ICT interventions. This research not only fills a critical gap in the systematic study of ICT applications in early childhood and primary education but also provides targeted evidence to inform educational policymakers and practitioners. By analyzing the role of predictors, the findings offer valuable theoretical insights and practical guidance for optimizing the design and implementation of educational technologies and advancing the digital transformation of basic education.

## 3 Research design

### 3.1 Research methods and tools

This study employs a meta-analysis approach to systematically synthesize recent research on the impact of ICT in early childhood and primary education. Key information, including sample sizes, means, and standard deviations from the included studies, was collected to calculate the overall effect size using the standardized mean difference (SMD) method. Heterogeneity was assessed using the I^2^ statistic. Depending on the degree of heterogeneity, either a random-effects model or a fixed-effects model was used to ensure the accuracy and robustness of the results. The use of the random-effects model is preferred when there is significant variability between studies, while the fixed-effects model is applied when studies are considered more homogenous.

Data analysis was conducted using StataSE, a statistical software designed for advanced meta-analyses. This tool was employed to calculate effect sizes, generate forest plots, funnel plots, and other visualizations. Throughout the research process, strict adherence to the PRISMA guidelines was maintained to ensure transparency in the inclusion and exclusion of studies and to maintain the replicability of the study.

### 3.2 Data sources

Comprehensive searches were conducted across multiple databases, including Web of Science, SCOPUS, ScienceDirect, Wiley Interscience, JSTOR, EBSCOhost, and CNKI. Using advanced search options, literature published between January 2014 and June 2024 was identified through a combination of English and Chinese keywords. The following keyword combinations were used in each database:

Keywords: (“Pre-Primary Education” OR “Preschool” OR “Young children” OR “Kindergarten” OR “Early Childhood Education”) AND (“Elementary Education” OR “Elementary School” OR “Elementary Student” OR “Primary Education” OR “Primary School” OR “Primary Student” OR “Pupils”) AND (“Information and Communication Technology” OR “Communication Technology” OR “Information Technology” OR “ICT” OR “Educational Technology” OR “Media”) AND (“Experimental” OR “Control” OR “Quasi-experimental” OR “Pre-test” OR “Post-test” OR “Pretest” OR “Posttest” OR “Pre test” OR “Post test”).

To ensure comprehensive coverage, supplementary searches were conducted on Google Scholar. This multi-database approach ensured that relevant studies were identified across both Western and Chinese-language research landscapes.

Table 1 outlines the key categories and their respective search terms. Boolean “AND” operators were used to combine terms across categories (e.g., Category 1 AND Category 2 AND Category 3 AND Category 4). This process ensured that all selected papers addressed early childhood education, primary education, ICT, and experimental research. Additionally, supplementary searches were conducted using Google Scholar to ensure comprehensive coverage.

**Table 1:** Search terms used in this Review.

Type	Category	Search terms
1	Early Childhood Education	Pre-Primary Education, Preschool, Young Children, Kindergarten, Early Childhood Education
2	Primary Education	Elementary Education, Elementary School, Elementary Student, Primary Education, Primary School, Primary Student, Pupils
3	ICT	Information and Communication Technology, Communication Technology, Information technology, ICT, Educational Technology, Media
4	Experimental Research	Experimental, Control, Quasi-experimental, Pre-test, Post-test, Pretest, Posttest, Pre test, Post test

### 3.3 Inclusion criteria

This study utilized the Population, Intervention, Comparison, Outcome, and Study Design (PICOS) framework alongside a review of article titles and abstracts to identify eligible studies (Eriksen and Frandsen, 2018). Research that met the following criteria was included:

Population: Participants were typically developing children aged 3–12 years, excluding those with special educational needs.

Intervention: The intervention involved the application of ICT in formal or informal learning environments.

Comparison: Studies included comparisons between experimental and control groups or employed pre-and post-test designs.

Outcome: Studies provided clear indicators of learning outcomes, such as changes in academic achievement, cognitive abilities, or social skills.

Study Design: Only randomized controlled trials (RCTs) or quasi-experimental studies were included, and they needed to report complete data for effect size extraction (e.g., means, standard deviations, and sample sizes).

Additionally, included studies had to be peer-reviewed publications written in English or Chinese, focused on the impact of ICT on preschool or primary school students, and published between 2014 and 2024.

The following studies were excluded:

1. Research involving middle or high school students in basic education.
2. Observational studies, monographs, conference papers, theoretical articles, literature reviews, or publications in languages other than English or Chinese.
3. Studies with sample sizes fewer than 10 participants or incomplete data reporting.

### 3.4 Standardized coding

**Figure 1:**
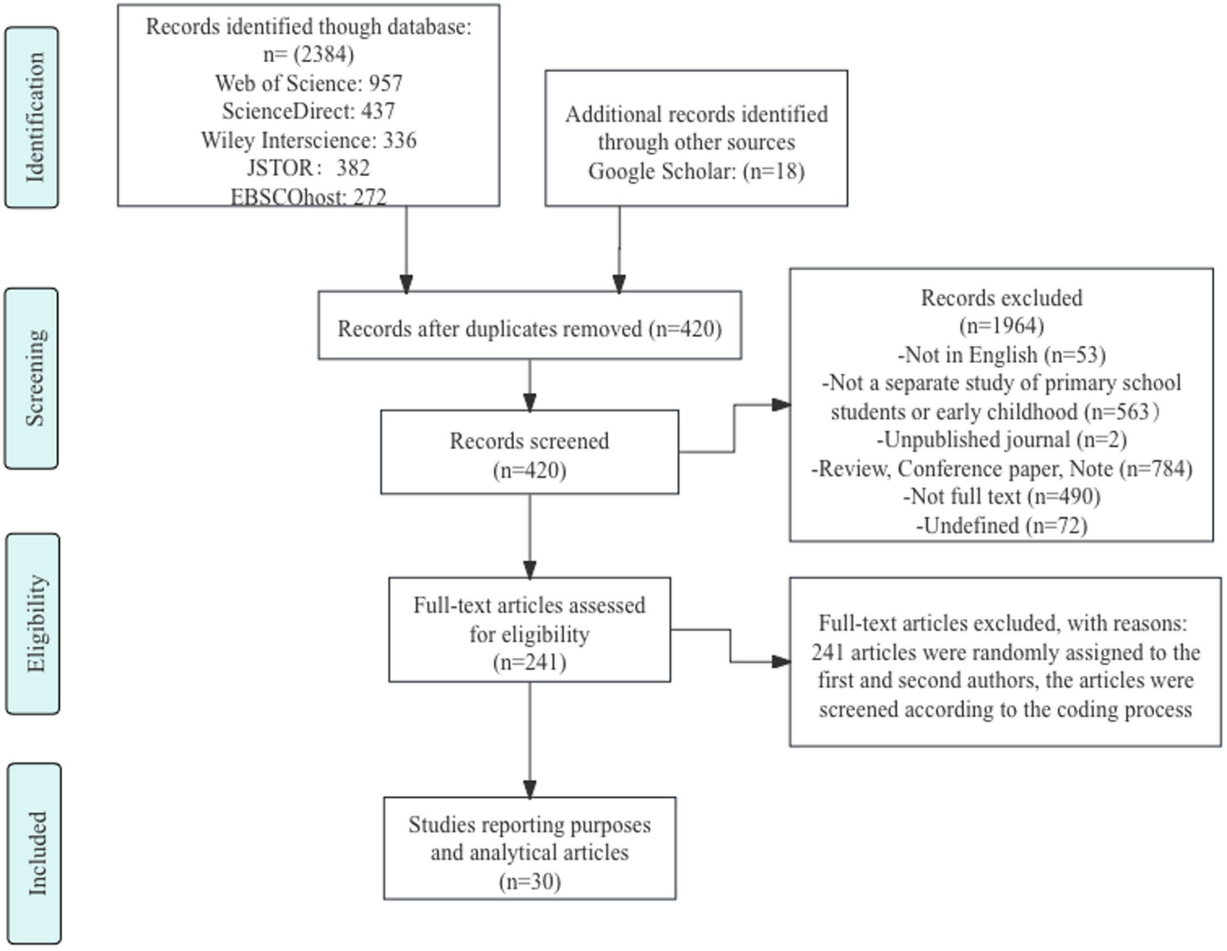
Literature review process.

**Figure 2:**
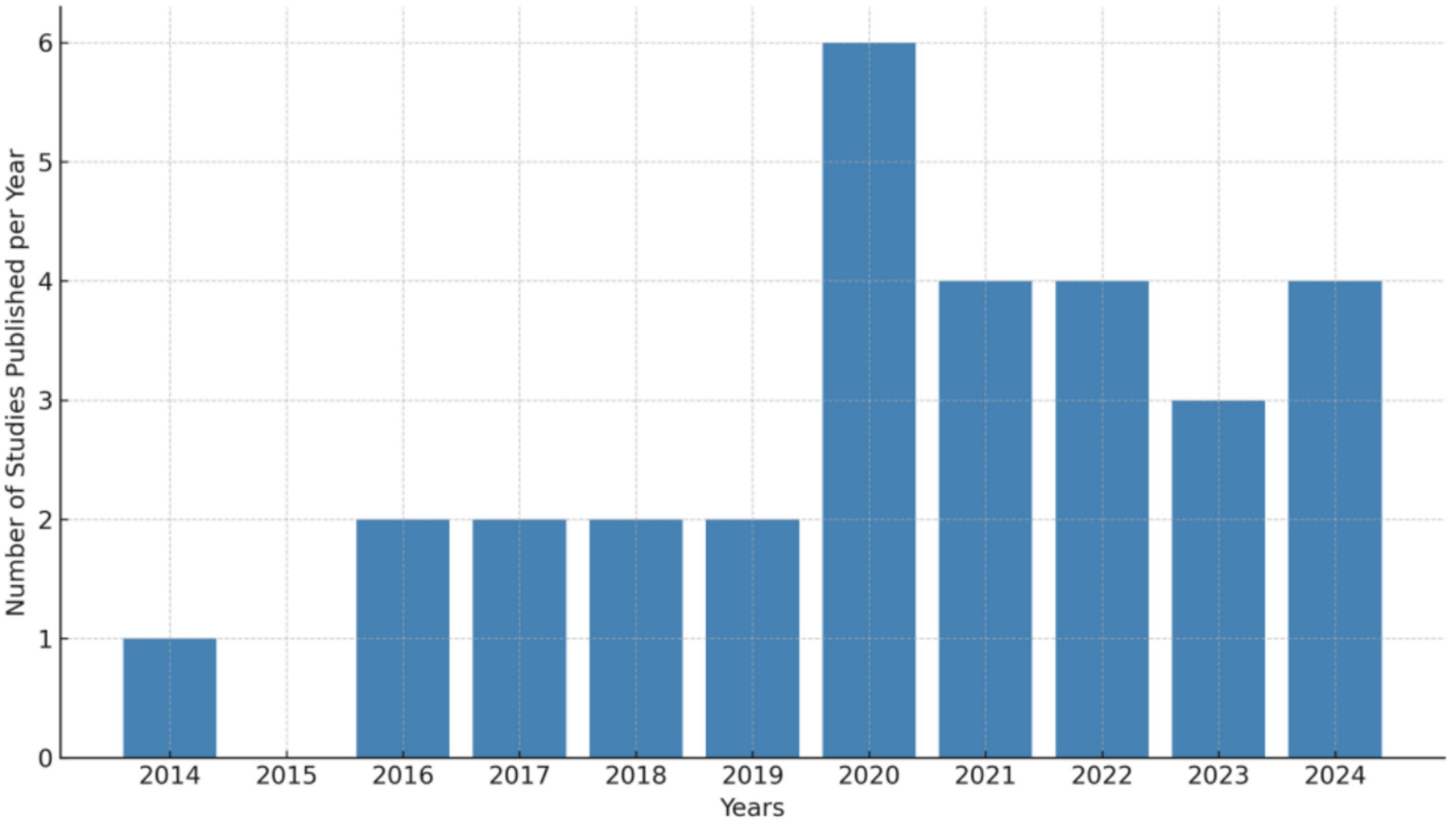
Located literature from 2014 to 2024.

Data extraction and coding for the meta-analysis were independently conducted by two researchers. The coded variables included participant information (e.g., age, sample size), intervention characteristics (e.g., application type, duration), and outcome measures (e.g., type of effect size, means, standard deviations). Any discrepancies were resolved through discussion or arbitration by a third researcher. Following coding, heterogeneity was assessed (e.g., using the I^2^ statistic), and a random-effects or fixed-effects model was chosen based on the results. The detailed literature selection process is shown in Figure 1, while the publication trend of relevant studies from 2014 to 2024 is illustrated in Figure 2.

Table 2 presents a comprehensive summary of the characteristics and variables coded from the studies included in this meta-analysis. It categorizes essential information such as publication details, study designs, participant demographics, and intervention characteristics. This table serves as a key reference for understanding the scope and diversity of the included studies, ensuring transparency and reproducibility in the coding process. Additionally, it provides a clear overview of the key features analyzed, which underpin the conclusions drawn from the study.

**Table 2:** Literature coding table included in the meta-analysis.

No	Author	Year	n_exp	n_ctrl	Area	Application type	Domain subject	Duration	Research type
1	Ihmeideh and Al-Maadadi (2018)	2018	48	48	Jordan	Touch screen	Language	6–18 weeks	Experimental
2	Zaranis (2016)	2016	165	170	Greece	Digital interactive	Subject knowledge	1–6 weeks	Quasi-experimental
3	Aladé et al. (2016)	2016	20	20	USA	Touch screen	Subject knowledge	≤1 week	Experimental
4	Neumann (2018)	2018	24	24	Australia	Touch screen	Language	6–18 weeks	Experimental
5	Vatalaro et al. (2018)	2018	31	32	USA	Touch screen	Language	6–18 weeks	Quasi-experimental
6	Furman et al. (2019)	2019	12	11	Argentina	Touch screen	Subject knowledge	1–6 weeks	Quasi-experimental
7	Outhwaite et al. (2019)	2019	305	156	UK	Digital interactive	Subject knowledge	6–18 weeks	Experimental
8	W. Liu et al. (2021)	2021	65	63	China	Digital interactive	Social and emotional development	6–18 weeks	Quasi-experimental
9	Amorim et al. (2022)	2022	199	152	Brazil	Touch screen	Language	6–18 weeks	Experimental
10	Rojas-Barahona et al. (2022)	2022	377	377	Chile	Touch screen	Subject knowledge	>18 weeks	Experimental
11	Saleh and Ahmed Althaqafi (2022)	2022	20	20	Saudi Arabia	Digital interactive	Language	1–6 weeks	Quasi-experimental
12	Akay and Cakir (2023)	2023	18	20	Turkey	Digital interactive	Social and emotional development	6–18 weeks	Quasi-experimental
13	Gao et al. (2024)	2024	26	25	China	Touch screen	Language	≤1 week	Experimental
14	Tazouti et al. (2024)	2024	345	192	France	Digital interactive	Subject knowledge	>18 weeks	Quasi-experimental
15	Peña et al. (2024)	2024	101	152	Chile	Touch screen	Language	>18 weeks	Experimental
16	Kim and Cho (2017)	2017	44	42	South Korea	Touch screen	Social and emotional development	6–18 weeks	Quasi-experimental
17	Volk et al. (2017)	2017	124	135	Slovenia	Touch screen	Subject knowledge	>18 weeks	Experimental
18	Arvanitaki and Zaranis (2020)	2020	27	19	Greece	Touch screen	Subject knowledge	≤1 week	Quasi-experimental
19	Zhang et al. (2020)	2020	32	33	China	Touch screen	Social and emotional development	≤1 week	Quasi-experimental
20	Danaei et al. (2020)	2020	18	16	Iran	Digital interactive	Language	≤1 week	Quasi-experimental
21	Chang et al. (2020)	2020	42	42	China	Digital interactive	Subject knowledge	≤1 week	Experimental
22	Yamaç et al. (2020)	2020	49	47	Turkey	Touch screen	Language	6–18 weeks	Experimental
23	Huang et al. (2020)	2020	20	20	China, Taiwan	Digital interactive	Subject knowledge	6–18 weeks	Quasi-experimental
24	Hooshyar et al. (2021)	2021	36	43	Estonia	Digital interactive	Subject knowledge	≤1 week	Experimental
25	Nicolaidou et al. (2021)	2021	20	21	Cyprus	Digital interactive	Social and emotional development	≤1 week	Quasi-experimental
26	Karakostantaki and Stavrianos (2021)	2021	20	20	Greece	Digital interactive	Social and emotional development	1–6 weeks	Experimental
27	Bang et al. (2023)	2023	507	481	USA	Digital interactive	Social and emotional development	>18 weeks	Experimental
28	S. Liu et al. (2023)	2023	40	40	China	Digital Interactive	Language	1–6 weeks	Quasi-experimental
29	Prados Sánchez et al. (2023)	2023	43	43	Spain	Digital interactive	Language	1–6 weeks	Quasi-experimental
30	Elma et al. (2024)	2024	26	25	Turkey	Digital interactive	Subject knowledge	1–6 weeks	Quasi-experimental

### 3.5 Statistical analysis

The primary analysis was conducted using the *I*^2^ statistic to assess heterogeneity across studies. Based on the level of heterogeneity, either a random-effects model or fixed-effects model was applied. The choice of model was justified by the variability in study designs and populations.

## 4 Data analysis and results

Given the variations in research themes, study durations, sample sizes, and application type across the selected studies, efforts were made to ensure consistency in data analysis. Information such as the mean scores, standard deviations, and sample sizes for both experimental and control groups was recorded. These data were used to calculate effect sizes, specifically Hedges’ *g*, as well as the weighted mean effect size and corresponding 95% confidence intervals. In addition, Q-tests and I^2^ statistics were performed to assess whether the effect sizes were influenced by any predictors (Borenstein, 2023). The heterogeneity of the included studies was assessed using a combination of *Q*-tests and *I*^2^ statistics. The results indicated a *Q*-value of 955.05 (*p* < 0.001) and an *I*^2^ value of 96.96% (>75%), suggesting a high degree of heterogeneity among the studies.

### 4.1 Publication bias assessment

**Figure 3:**
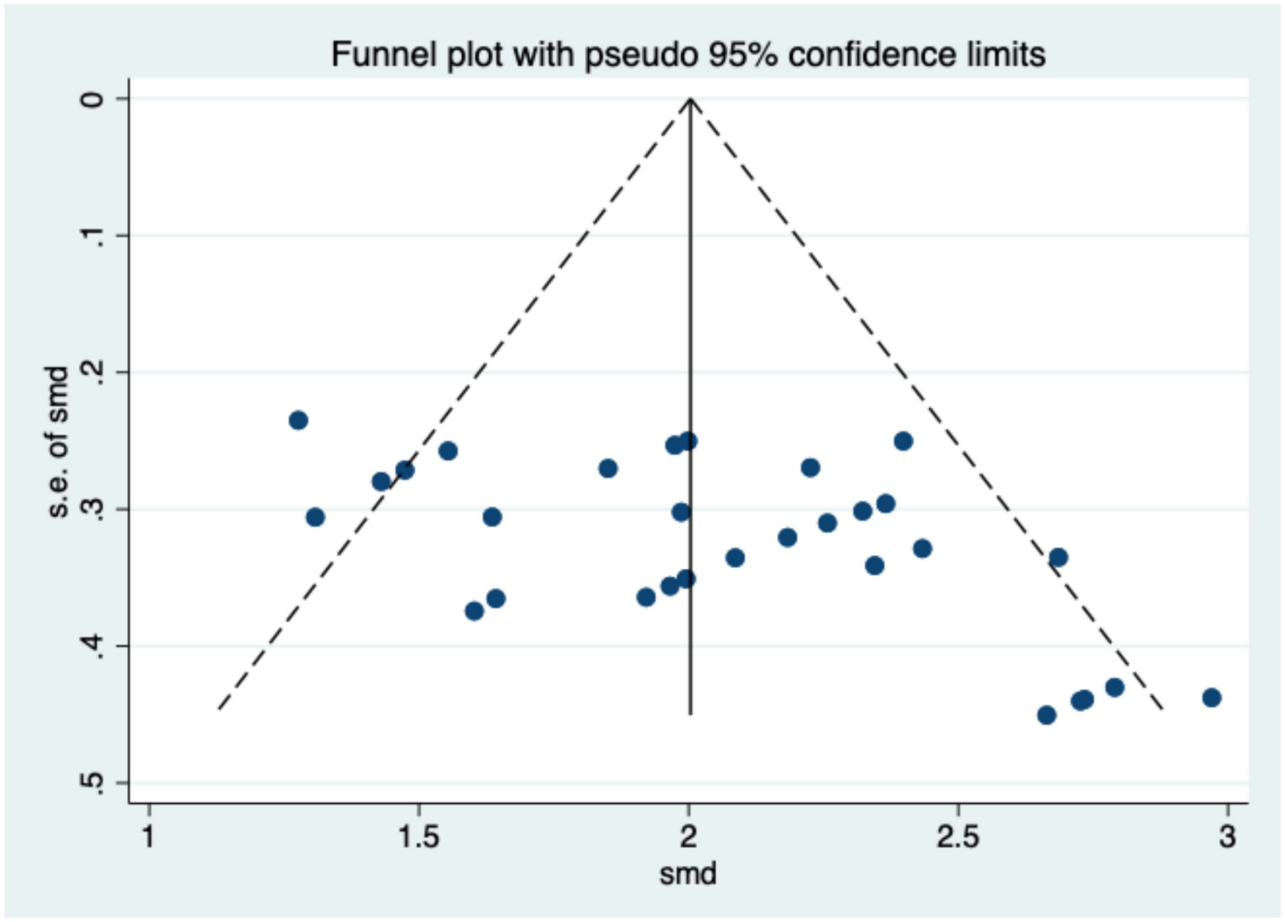
Funnel plot for publication bias test.

The assessment of publication bias aims to evaluate and detect the presence of bias in meta-analyses or systematic reviews, which may skew results by overrepresenting positive or significant findings. Specifically, studies with statistically significant or favorable outcomes are more likely to be published, while those with non-significant or negative results may remain unpublished, potentially affecting the overall conclusions of the analysis. Common methods for detecting publication bias include the Funnel Plot, Egger’s Regression Test, Begg and Mazumdar’s Test, the Trim and Fill Method, and the Fail-safe N Test. This study employed a combination of the Funnel Plot and Egger’s Regression Test to assess publication bias. As shown in Figure 3, the plot exhibits a symmetrical inverted funnel shape, with data points evenly distributed on both sides of the average effect size, indicating the absence of significant publication bias. The results of Egger’s Regression Test further support this conclusion (*t* = −0.43, n.s.). These findings confirm the absence of notable publication bias in the research field, enhancing the stability and reliability of the meta-analytic results.

### 4.2 Overview of research papers

All the peer-reviewed academic papers included in this study utilized standardized testing. Among these, 14 studies employed experimental designs, while 16 studies utilized quasi-experimental designs. In experimental designs, participants were randomly assigned to either the treatment group or the control group, whereas in the quasi-experimental studies, participants were not randomly assigned (Miller et al., 2020). Table 3 provides detailed information on the types of tests conducted in the peer-reviewed academic papers selected for this study.

**Table 3:** Type of tests conducted in reviewed papers.

Type of tests	Quantity
Experimental	14
Quasi-Experimental	16
Total	30

Table 4 presents the frequency of studies conducted across different geographic locations. The table provides a detailed breakdown of the distribution of studies in regions such as North America, Europe, Asia, Africa, and Oceania. Analyzing these data reveals clear insights into the level of involvement and activity in the field of educational technology research across various regions. The results indicate that research on the application of ICT in early childhood and primary education is predominantly concentrated in East Asia (e.g., China), North America (e.g., the United States), and parts of Europe (e.g., Greece and Turkey), with fewer studies originating from Africa, Southeast Asia, and South America. This uneven distribution may reflect disparities in research infrastructure, technological access, and national education policies.

**Table 4:** Frequency of research conducted at different geographic locations.

Geo-spatial coverage	Frequency of publications	%
China (including Taiwan)	6	20%
United States	3	10%
Greece	3	10%
Turkey	3	10%
Chile	2	6.67%
Australia	1	3.33%
South Korea	1	3.33%
United Kingdom	1	3.33%
France	1	3.33%
Spain	1	3.33%
Saudi Arabia	1	3.33%
Jordan	1	3.33%
Brazil	1	3.33%
Slovenia	1	3.33%
Argentina	1	3.33%
Estonia	1	3.33%
Cyprus	1	3.33%
Iran	1	3.33%

### 4.3 Examination of the impact of educational technology on preschool and primary school students

To investigate whether the use of information technology benefits preschool and primary school students, and whether the impact differs due to factors such as the type of technology, the subject areas involved, or the duration of the interventions, this study examines both the overall effects of educational technology on these students and the influence of several predictors (application type, domain subject, and intervention duration).

#### 4.3.1 Predictor analysis

The following is a description of each predictor considered in this study:

1. Application Type: This variable examines whether the impact of educational technology on learning outcomes varies by the type of technological interface used. Applications were categorized into two groups based on their primary mode of interaction and instructional design: (1) Touch Screen – referring to technologies that rely primarily on tactile interaction, such as tablets, smartphones, and touch-enabled devices. (2) Digital Interactive – referring to immersive or multimodal digital environments that promote active engagement through features like simulation, animation, or networked interaction. This includes tools such as augmented reality (AR), virtual reality (VR), Web 2.0 platforms, mobile media applications, digital games, and e-books. In cases where the same device (e.g., tablet) could fall under both categories, classification was based on the primary educational function described in the original study.
2. Domain Subject: The U. S. National Education Goals Panel defines children’s developmental outcomes in terms of physical and motor development, social and emotional development, learning quality, language development, and cognitive and foundational knowledge (Hudson and Willoughby, 2021). The literature reviewed in this study focuses primarily on language, subject knowledge, and social–emotional development. The purpose of considering domain subjects is to determine the relative learning outcomes across different subject areas, such as general subjects (when technology is used for learning multiple subjects), language, mathematics, music, science, and the arts.
3. Intervention Duration: This variable assesses the effect of different intervention durations on students’ relative learning outcomes. The duration of the interventions is categorized as follows: ≤ 1 week, 1–6 weeks, 6–18 weeks, and >18 weeks. The classification of intervention duration into the categories of ≤1 week, 1–6 weeks, 6–18 weeks, and >18 weeks was based on both the distributional characteristics of the included studies and practical educational considerations. Specifically, the 18-week threshold approximates the duration of a typical instructional semester in many educational systems, which usually ranges from 18 to 20 weeks excluding exam periods. This grouping scheme allowed for meaningful comparisons among short-term, medium-term, and long-term ICT interventions, while maintaining a balanced number of studies across categories to support statistical power.

#### 4.3.2 Overall effect size test

The overall effect of ICT on preschool and primary school children is presented in Table 5. The pooled effect size was 0.45 (*p* < 0.001) with a 95% CI [0.41, 0.49]. Hedges’ *g* is a form of standardized mean difference (SMD) that adjusts for small sample bias and is widely used in meta-analyses. According to Cohen’s (1988) statistical theory for effect sizes: an SMD between 0 and 0.2 indicates a small effect, between 0.2 and 0.5 indicates a moderate effect, between 0.5 and 1 indicates a substantial effect, and an SMD greater than 1 indicates a very large effect. The effect size observed in this study falls between 0.2 and 0.5, suggesting that the overall impact of educational technology on preschool and primary school children is moderate and positive.

**Table 5:** Educational technology’s overall effect on preschool and primary school children.

Effect Model	95% Confidence Interval (95% CI)					Heterogeneity Test			
	Number of effects	Effect size (SMD)	Standard error (SE)	Lower bound	Upper bound	Q-statistic	df	*P*	*I* ^2^
Random-effects model	30	0.45	0.018	0.41	0.49	955.05	29	<0.001	96.96%

#### 4.3.3 Influence of different predictors on the learning outcomes of preschool and primary school students

##### 4.3.3.1 Domain subject

**Figure 4:**
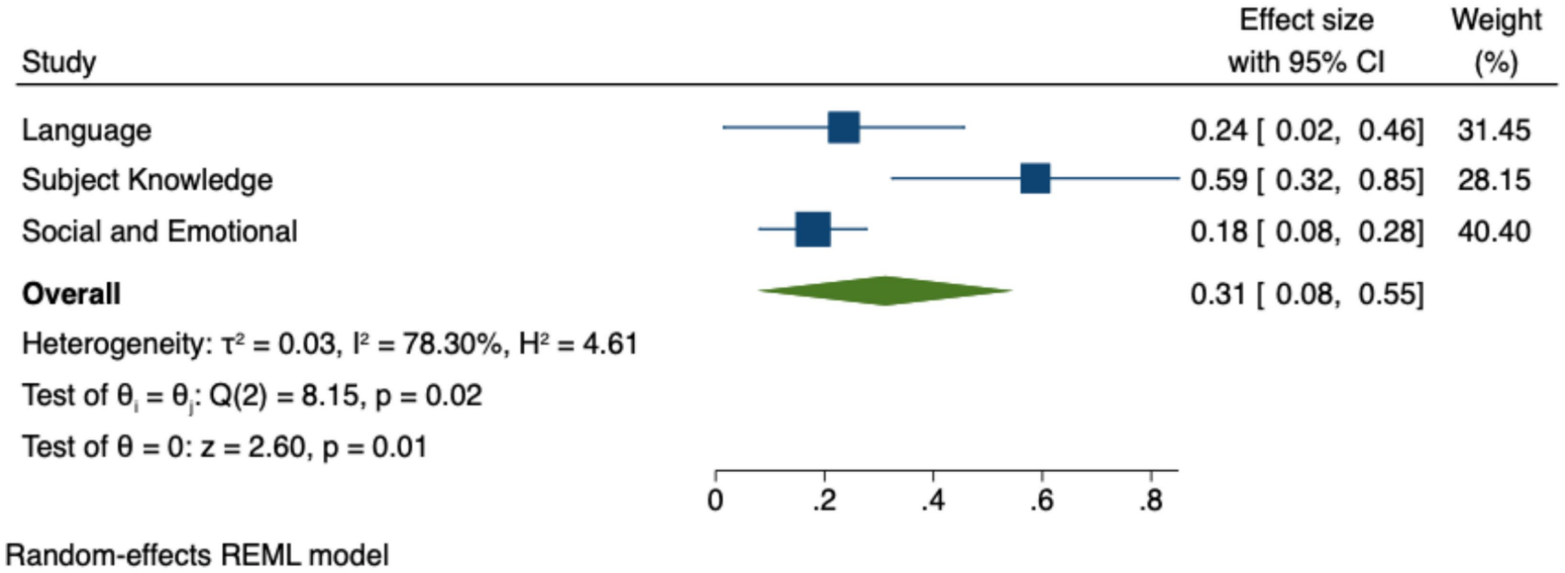
Effect sizes by domain subject.

To conduct the predictor analysis, studies were categorized by subject domain. Table 6 presents the Hedges’ *g* estimates, *Z* values, *p*-values, and confidence intervals for each domain. Figure 4 illustrates the 95% confidence intervals for the effect sizes of different domains. The results indicate that Language (g = 0.24, *p* < 0.001) and Subject Knowledge (*g* = 0.59, *p* < 0.001) show larger effects, whereas Social and Emotional Development (*g* = 0.18, *p* < 0.05) demonstrates a smaller effect. These findings suggest that technology has a moderate impact on language and subject knowledge development, while its impact on social and emotional development is relatively weaker. Additionally, Figure 4 highlights that the confidence intervals for Language and Subject Knowledge show more pronounced and tightly clustered around the effect sizes, indicating more robust and consistent effects across studies, while the confidence interval for Social and Emotional Development indicates a smaller effect, though still statistically significant. This implies that educational technology is more effective in enhancing core academic learning outcomes, with comparatively weaker effects on social and emotional development.

**Table 6:** Predictor analysis by domain subject.

Domain subject	Language	Subject knowledge	Social and emotional development
Number of samples (k)	11	12	7
Total sample size (N)	1,198	2,709	1,386
Effect size	0.24	0.59	0.18
Z	2.10	4.39	3.58
P (effect size)	<0.05	<0.001	<0.05
95% upper	0.46	0.85	0.28
95% lower	0.02	0.32	0.08

##### 4.3.3.2 Application type

**Figure 5:**
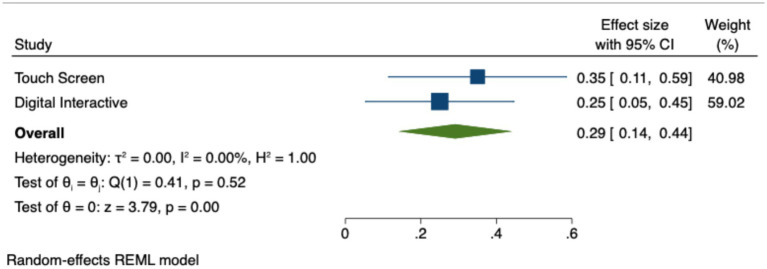
Effect sizes by application type.

The studies were grouped by type of technological application for predictor analysis. The results of the analysis reveal that different technological devices have varying impacts on students. Figure 5 presents the 95% confidence intervals for the effect sizes of the two types of application. Table 7 provides the Hedges’ *g* values, *p*-values, and confidence intervals. Specifically, the effect sizes for touchscreen (*g* = 0.35, *p* < 0.01) and digital interactive (*g* = 0.25, *p* < 0.05) were both significant, indicating a moderate positive impact on students, which is statistically significant. From Figure 5, it is evident that the effect size for touchscreen is consistently larger, with the confidence interval indicating a more robust effect compared to digital interactive. Table 7 further supports this by presenting the effect sizes, Z-values, p-values, and confidence intervals for both application types. These results suggest that touchscreen technologies provide stronger, more interactive benefits for students, likely due to their more hands-on, engaging nature, while digital interactive technologies still offer significant but somewhat less pronounced effects.

**Table 7:** Predictor analysis by application type.

Application type	Touch screen	Digital interactive
Number of samples (k)	14	16
Total sample size (N)	2,231	3,062
Effect Size	0.35	0.25
Z	2.92	2.50
P (effect size)	<0.01	<0.05
95% upper	0.59	0.45
95% lower	0.11	0.05

##### 4.3.3.3 Intervention duration

**Figure 6:**
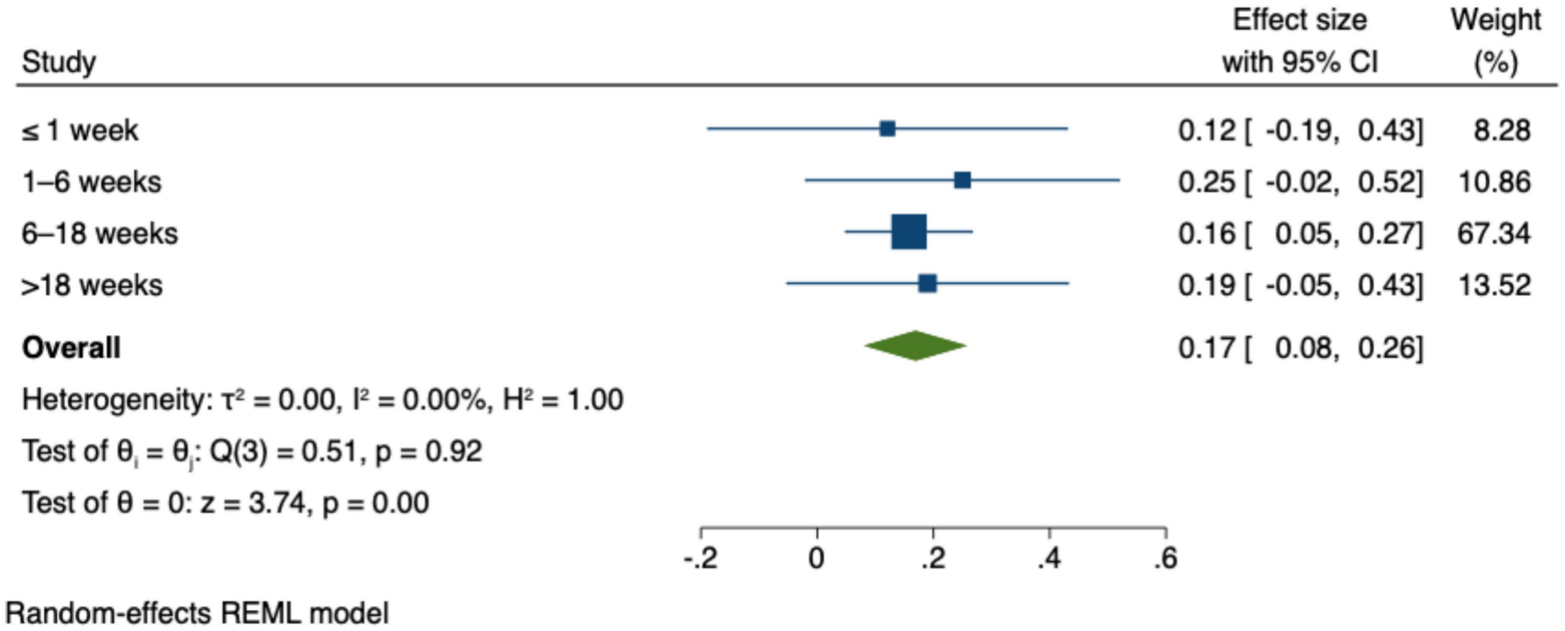
Effect sizes by intervention duration.

To conduct the analysis of predictors, the studies included in the review were categorized into different experimental durations. Table 8 presents the effect size estimates, *Z*-values, *p*-values, and confidence intervals for all experimental durations. Figure 6 illustrates the 95% confidence intervals for the effect sizes across these experimental durations. The results indicate that only the duration of “6–18 weeks” (*g* = 0.16, *p* < 0.01) was statistically significant. The results indicate that only the “6–18 weeks” condition produced a statistically significant effect, as its 95% confidence interval did not include zero. In contrast, the durations of “≤1 week” (*g* = 0.12, n.s.), “1–6 weeks” (*g* = 0.25, n.s.), and “>18 weeks” (*g* = 0.19, n.s.) were not statistically significant, as their confidence intervals crossed zero. These results suggest that both very short and extended intervention durations may not fully capture the potential benefits of ICT use in early education. The findings from Figure 6 reinforce the interpretation that moderate-length interventions—such as those lasting 6–18 weeks—may be more effective in producing measurable outcomes. This could be due to factors such as insufficient engagement time in shorter interventions and declining novelty or participant fatigue in longer ones.

**Table 8:** Predictor analysis by intervention duration.

Intervention duration	≤1 week	1–6 week(s)	6–18 weeks	>18 weeks
Number of samples (k)	8	7	10	5
Total sample size (N)	440	655	1,407	2,791
Effect Size	0.12	0.25	0.16	0.19
Z	0.78	1.83	2.88	1.53
P (effect size)	n.s.	n.s.	<0.01	n.s.
95% upper	0.43	0.52	0.27	0.43
95% lower	−0.19	−0.02	0.05	−0.05

These findings suggest that moderate-length intervention durations are more effective in capturing the impact of ICT on student learning outcomes. Shorter or longer durations may fail to fully harness the technology’s potential, likely due to factors such as initial novelty or diminishing engagement over time. This conclusion aligns with existing literature emphasizing the critical role of selecting an appropriate intervention duration to ensure reliable effect size measurements (Morris, 2008). However, it diverges from other perspectives in the literature, which argue that shorter intervention periods—often considered more controllable and less prone to extraneous variables—tend to yield more pronounced effects than longer-term interventions (Golos et al., 2013).

## 5 Discussion

This study employs meta-analytic techniques to systematically synthesize findings from 30 experimental and quasi-experimental studies investigating the integration of ICT in early childhood and primary education. The analysis evaluates the overall effectiveness of ICT on students’ learning outcomes and developmental progress, while examining the moderating influences of three key variables: application type, domain subject, and intervention duration. Based on the synthesized data and subsequent analysis, several significant conclusions are drawn:

### 5.1 The overall positive impact of ICT across time and regions

This section addresses the first research objective: to synthesize and integrate existing literature evaluating the impact of ICT on young children and primary students, with attention to the distribution of studies across geographic regions and publication years (see Table 4). Figure 2 illustrates the publication trends of studies on the application of educational technology in primary and early childhood education from 2014 to 2024. The data reveals a significant increase in publications during 2019–2024, with 23 studies published, compared to only 7 studies between 2014 and 2019. This sharp growth reflects the increasing attention that educational technology has garnered in the fields of early childhood and primary education. Several factors may account for this rise. The widespread adoption of digital devices, such as tablets and interactive whiteboards, in both homes and schools has expanded opportunities for the integration of educational technology. Moreover, young children and primary school students now frequently engage with these tools in both formal and informal learning environments. Additionally, the global COVID-19 pandemic has further accelerated the integration of educational technology into home and school settings, driving the widespread adoption of digital teaching practices.

As discussed in the introduction, the application of ICT in early childhood and primary education has been a subject of academic debate. Despite some studies reporting potential negative or null effects of ICT (Dong and Newman, 2016; Kerckaert et al., 2015), the overall meta-analysis revealed a moderate and significant positive effect (*g* = 0.45, *p* < 0.001), indicating that ICT use in early and primary education generally promotes children’s learning and development. This overall effect supports the theoretical potential of ICT to enhance educational outcomes across diverse learner populations. While this study confirms the general efficacy of ICT, the magnitude and focus of its impact may vary across different learning domains and instructional contexts, which are further explored in the following sections.

Additionally, this study comprehensively examined the role of ICT across different dimensions of learning and development. The results showed that ICT has a significant positive impact on language development, subject knowledge acquisition, as well as social–emotional development and emotional regulation. Notably, ICT’s effect on language skills and subject knowledge was particularly pronounced. This finding aligns with the research of scholars such as Hussain (2018), Simbolon et al. (2020), and Weber and Greiff (2023), who have concluded that the use of ICT effectively promotes language development, subject understanding, and emotional regulation in young children and primary students. For example, ICT tools enhance children’s language expression and vocabulary acquisition through interactive learning resources and multisensory stimuli, while online learning platforms and virtual laboratories facilitate the mastery of complex subject concepts (Nikolopoulou et al., 2019). Moreover, in the realm of social–emotional development, the application of ICT through gamified learning and virtual interactive environments provides rich opportunities for emotional expression and communication, fostering social skills and emotional awareness (Melo-Solarte and Díaz, 2018). Thus, ICT holds significant potential in the early and primary education stages, offering new pathways for promoting multidimensional learning and holistic development (Weber and Greiff, 2023). Therefore, it can be concluded that ICT has substantial potential for the development of diverse abilities in young children and primary school students, and it’s appropriate use in early and primary education can facilitate the development of these diverse capabilities (Kerckaert et al., 2015; Xiao et al., 2019).

### 5.2 Differential impact of various technology devices on young children and primary school students

This section responds to the second research objective by examining how the type of ICT application influences learning outcomes. The application of digital interactive technologies (such as augmented reality (AR), virtual reality (VR), Web 2.0, mobile media apps, and digital games) and touchscreen devices (such as tablets, smartphones, and e-readers) in educational settings each possesses unique characteristics. Digital interactive technologies provide immersive environments that transcend temporal and spatial limitations, enabling learners to intuitively grasp complex concepts (Borba et al., 2018). Their flexibility and autonomy create novel opportunities for student engagement. In contrast, touchscreen devices, with their user-friendly interfaces and lower cost, foster independent exploration, making them widely adopted in educational contexts.

These technologies have been implemented in varied educational contexts, each producing distinct learning effects. The findings suggest that touchscreen devices, compared to non-touchscreen alternatives, offer greater interactivity, stimulating curiosity and fine motor skills in students (Booton et al., 2021; Liu and Hwang, 2021). For preschoolers, touchscreen devices support exploratory learning and facilitate the application of knowledge in transferable ways (Wang et al., 2021). In primary education, hey enhance concentration, sustain interest, and contribute to improved learning outcomes (Guan et al., 2022). Digital interactive technologies, on the other hand, provide immersive, multisensory environments that help learners intuitively grasp abstract or complex concepts. These results suggest that both application types can effectively support children’s development, albeit through different mechanisms and cognitive pathways (Brucker et al., 2021; Samuelsson et al., 2021).

### 5.3 Positive impact of ICTs different subject domains, with significant effects in language and subject knowledge domains

To further address the second research objective, this section explores the effect of ICT across different subject domains. Analysis of 30 studies revealed that, compared to research in the socio-emotional domain, scholars are more inclined to investigate the effects of ICT on language skills and subject knowledge acquisition. This research trend may be driven by several factors. One reason, as suggested by this study, is that language skills and subject knowledge are frequently encountered in students’ daily academic activities, and their learning outcomes are more easily quantifiable and assessable. The development of language skills and mastery of subject knowledge directly impact teaching quality and students’ learning abilities, offering a clearer reflection of the practical effects of educational activities. Additionally, such studies provide strong practical guidance for teachers, offering insights into optimizing teaching methods, enhancing classroom efficiency, and innovating instructional strategies (Liu et al., 2022; Sepp et al., 2022). This not only addresses the practical needs of educators but also provides a theoretical basis for the further integration and application of ICT in education. Therefore, the concentration of research in language and subject knowledge domains reflects both the practical concerns of education research and its high adaptability to educational practice. However, this focus may have led to a relative neglect of research in the socio-emotional domain, suggesting that future studies should balance academic and practical concerns while also addressing the socio-emotional aspects of education (Lozano-Peña et al., 2021).

### 5.4 Variations in the impact of different experimental durations on student learning outcomes: longer interventions do not always yield better results

Lastly, this section examines intervention duration as a predictor of ICT effectiveness, thereby completing the analysis of the second research objective. In this study, intervention duration refers specifically to the total length of time (in weeks) during which ICT tools were formally integrated into instructional activities as part of an educational intervention. This should not be confused with daily screen time, which measures the total hours children spend using digital devices, nor with general technology use duration, which may include non-instructional digital exposure.

The meta-analysis results indicate that ICT interventions lasting 6 to 18 weeks yield the most favorable outcomes for young children and primary school students. In contrast, both shorter (≤1 week, 1–6 weeks) and longer (>18 weeks) durations did not produce statistically significant effects.

This pattern does not necessarily imply that ICT becomes ineffective over time. Rather, it may reflect several factors. First, the novelty effect may fade, reducing learners’ motivation and engagement. Second, longer interventions often face implementation challenges, such as limited teacher capacity and insufficient high-quality, child-specific digital resources. These issues hinder the adaptive alignment of ICT tools with evolving instructional content and goals. Without continuous adaptation, learning gains may plateau, and students may experience fatigue. Additionally, the small number and methodological heterogeneity of studies in the >18-week group may have reduced the precision of the estimated effects.

Therefore, while ICT shows clear short-to medium-term benefits, sustaining its long-term impact requires ongoing pedagogical adaptation, dynamic content updates, and strengthened teacher training in ICT integration. These findings highlight the need for future research on effective strategies to maintain ICT engagement and effectiveness across extended instructional periods (Bentri and Hidayati, 2023).

## 6 Limitations and directions for future research

In 2012, the American Early Childhood Education Association published a groundbreaking statement on the use of technology and interactive media in early childhood and primary education (Cochran et al., 2012). This statement underscores the importance of intentional and appropriate use of technology and interactive media as effective tools to support children’s learning and development. It introduces “developmentally appropriate practices” as the foundational principle for integrating educational technology in preschool and primary education. By advocating for the timely and context-sensitive application of technology, the statement emphasizes alignment with children’s cognitive developmental stages and fundamental needs, thereby promoting holistic development.

In practice, both preschool and primary school teachers, as well as parents, need to shift their views on the use of information technology in education. While the impact of ICT on cognitive development, particularly in intellectual areas, should be a focal point, attention must also be given to its role in fostering children’s diverse capabilities, promoting their physical and mental health, and ensuring their holistic development. As noted earlier, the effects of ICT differ across various educational domains. Therefore, when selecting technologies for the classroom, educators should tailor their choices based on the specific teaching content and the students’ individual needs, applying educational technology in a flexible manner. In preschool and early primary education, teaching should primarily focus on hands-on, game-based activities that directly engage children with learning content to facilitate cognitive development. In upper primary grades, technologies should be used to help students understand learning materials in a more visual and intuitive manner and to apply the knowledge learned across subjects and in real-life contexts.

Regarding the duration of technology use, teachers should exercise caution. If the duration is too short, it may not be sufficient for children to grasp the content or to engage their learning motivation. On the other hand, prolonged use may lead to a decline in interest and may have negative effects on students’ vision, especially with excessive screen time. In 2019, the World Health Organization (WHO) issued screen time guidelines suggesting that children under the age of 2 should avoid screen exposure entirely. For children aged 2–5, screen time should be limited to 1 h per day, ideally with high-quality, interactive programming watched together with a caregiver. For children aged 5–12, the WHO did not specify exact screen time limits but emphasized the importance of balancing sedentary activities with adequate physical activity and sleep (World Health Organization, 2019). Thus, teachers must strictly regulate the duration of ICT interventions to optimize teaching outcomes.

Based on the results of the meta-analysis, it appears that the impact of ICT on socio-emotional development is still lagging behind that in other areas. While ICT has shown positive effects in promoting language, subject knowledge, and cognitive abilities, its impact on emotional understanding, emotional regulation, and social skills development has been less pronounced than expected. Socio-emotional skills are central to children’s overall development, directly influencing their interpersonal relationships and social adaptation (Alwaely et al., 2020). Therefore, future applications of educational technology should focus on integrating emotional education, developing resources that incorporate emotional interaction and social scenario simulations to further enhance the holistic development of children’s socio-emotional capabilities.

Although this meta-analysis offers important insights into the role of ICT in early childhood and primary education, several limitations must be acknowledged. The potential for publication bias exists, as studies with significant results are more likely to be published. In addition, the heterogeneity across studies—due to differences in sample characteristics, ICT tools, and intervention duration—limits the consistency of the findings. Furthermore, the generalizability of the results is constrained by the specific educational and cultural contexts of the studies.

To address these limitations, future research should focus on several key areas. First, longitudinal studies are needed to assess the long-term effects of ICT on children’s cognitive and socio-emotional development. These studies would provide valuable insights into how ICT interventions influence learning outcomes over time. Second, cross-cultural and cross-regional studies should be conducted to examine how contextual factors—such as variations in educational systems, cultural attitudes toward technology, and access to resources—affect the effectiveness of ICT in different settings. Furthermore, research should explore the development of ICT tools specifically targeting socio-emotional learning, integrating emotional education, social interactions, and real-world simulations to enhance children’s emotional and social skills. Another important direction for future research is to investigate the impact of specific ICT modalities, such as AR, VR, or tablet-based applications, on distinct developmental domains. Lastly, indirect effects, such as ICT’s influence on children’s motivation, self-regulation, and attention, should be explored to understand the broader impact of technology in early education.

Addressing these gaps will provide a more comprehensive understanding of how ICT can be effectively integrated into educational settings, ensuring that digital tools support all aspects of children’s development.
